# Bilateral fibular fractures in a pre-ambulant infant

**DOI:** 10.1007/s00247-020-04738-6

**Published:** 2020-07-03

**Authors:** Michael Paddock, David Horton, Amaka C. Offiah

**Affiliations:** 1 Medical Imaging Department, Barnsley Hospital NHS Foundation Trust, Gawber Road, Barnsley, UK; 2 Academic Unit of Child Health, Sheffield Children’s NHS Foundation Trust, University of Sheffield, Damer Street Building, Western Bank, Sheffield, S10 2TH UK; 3 Department of Radiology, Hull University Teaching Hospitals NHS Trust, Hull, UK; 4 Department of Radiology, Sheffield Children’s NHS Foundation Trust, Western Bank, Sheffield, UK

**Keywords:** Fibula, Fracture, Infant, Inflicted injury, Nonaccidental injury, Radiography, Suspected physical abuse

## Abstract

Multiple long-bone fractures, particularly bilateral fractures, are of moderate specificity for inflicted injury (physical abuse) in infants and young children. Bilateral healing fractures of the fibulae are rare and, depending on age, raise the suspicion of inflicted injury. We report healing undisplaced fractures of both fibulae, in almost identical positions, in a pre-ambulant infant. The caregivers reported that the infant repeatedly banged his legs against the metal frame of his playpen. A video of this mechanism was provided to the instructed radiology expert and showed that the point of impact of the infant’s legs against the metal frame was at a similar level to the radiographic abnormalities. This mechanism was therefore believed to be consistent with the injuries, resulting in a diagnosis of self-inflicted bilateral fibular fractures and not of inflicted injury.

## Introduction

Inflicted injury (also termed non-accidental injury) is more common in infants and young children under the age of 2 years, in particular in those younger than 12 months old [1]. Multiple long-bone fractures, specifically those which are bilateral, are moderately specific for physical abuse. Bilateral healing fibular fractures are rare; they are said to be non-specific injuries, indicative of indirect forces, but usually indicate inflicted injury (physical abuse) when associated with other injuries [2], particularly in pre-ambulant infants. We present a case of self-inflicted healing fractures of both fibulae in a 6-month-old pre-ambulant infant, confirmed by video evidence.

## Case report

A 6-month-old boy was presented to his general practitioner after his caregivers noticed that he was not holding or using his left leg in a normal manner. He was given a diagnosis of transient synovitis and discharged home. His caregivers persistently sought medical attention for the limited use of his left leg: He was presented to the emergency department 3 days later, and again 6 days after his initial presentation to his general practitioner. No other concern was reported and there was no relevant medical history of note. The clinical teams found nothing suspicious in the caregivers’ behaviour or social history. There was no history of illicit drug or excessive alcohol use, the family was not previously known to social services, and the boy’s immunisations were up to date. The child was well cared for and there were no bruises, scratches or other stigmata of abuse.

**Fig. 1:**
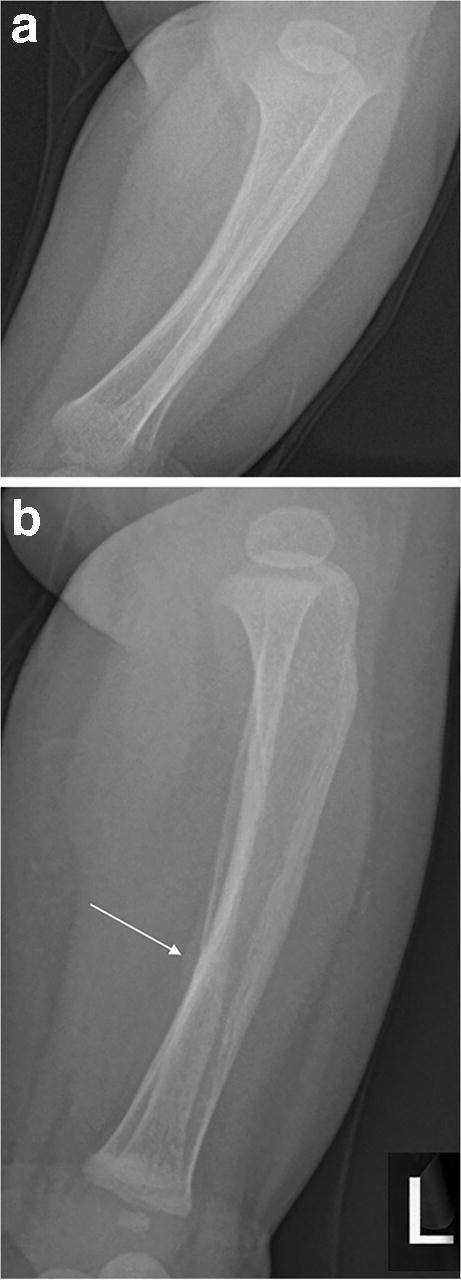
A 6-month-old boy with healing fractures of both fibulae. **a–b** Anteroposterior (**a**) and lateral (**b**) radiographs (cropped and magnified) of the left tibia and fibula. There is subtle periosteal reaction along the posterior cortex of the fibular shaft at the junction of middle and distal thirds (*arrow*). A faint horizontal line runs through the posterior cortex of the fibula, centred on the periosteal reaction and suggests an undisplaced fracture. Images were initially reported as normal

**Fig. 2:**
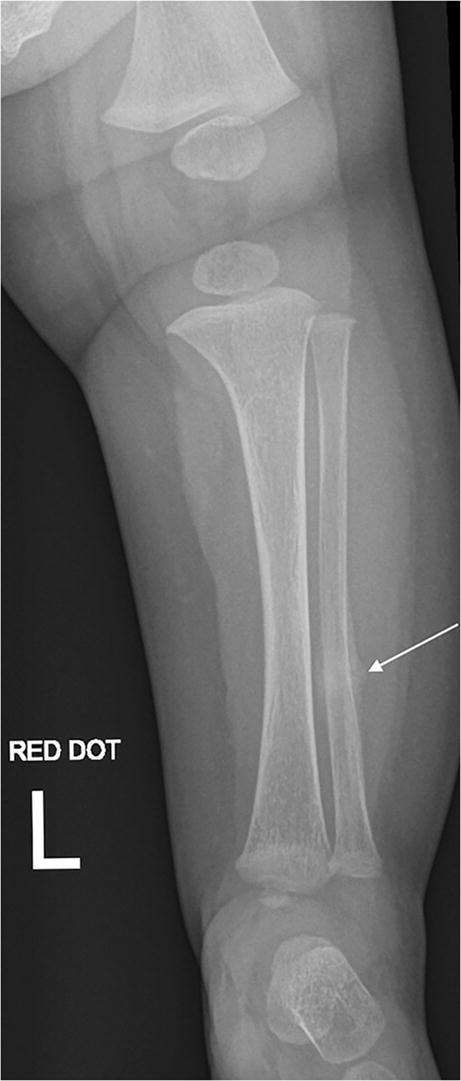
An anteroposterior radiograph of the left tibia and fibula was performed 2 days after Fig. 1, repeated due to persistent symptoms and initial “normal” radiographs. There is increased periosteal reaction (*arrow*) compared to the previous radiographs, consistent with progressive healing of an undisplaced fracture. There is normal density and modelling of the imaged bones

**Fig. 3:**
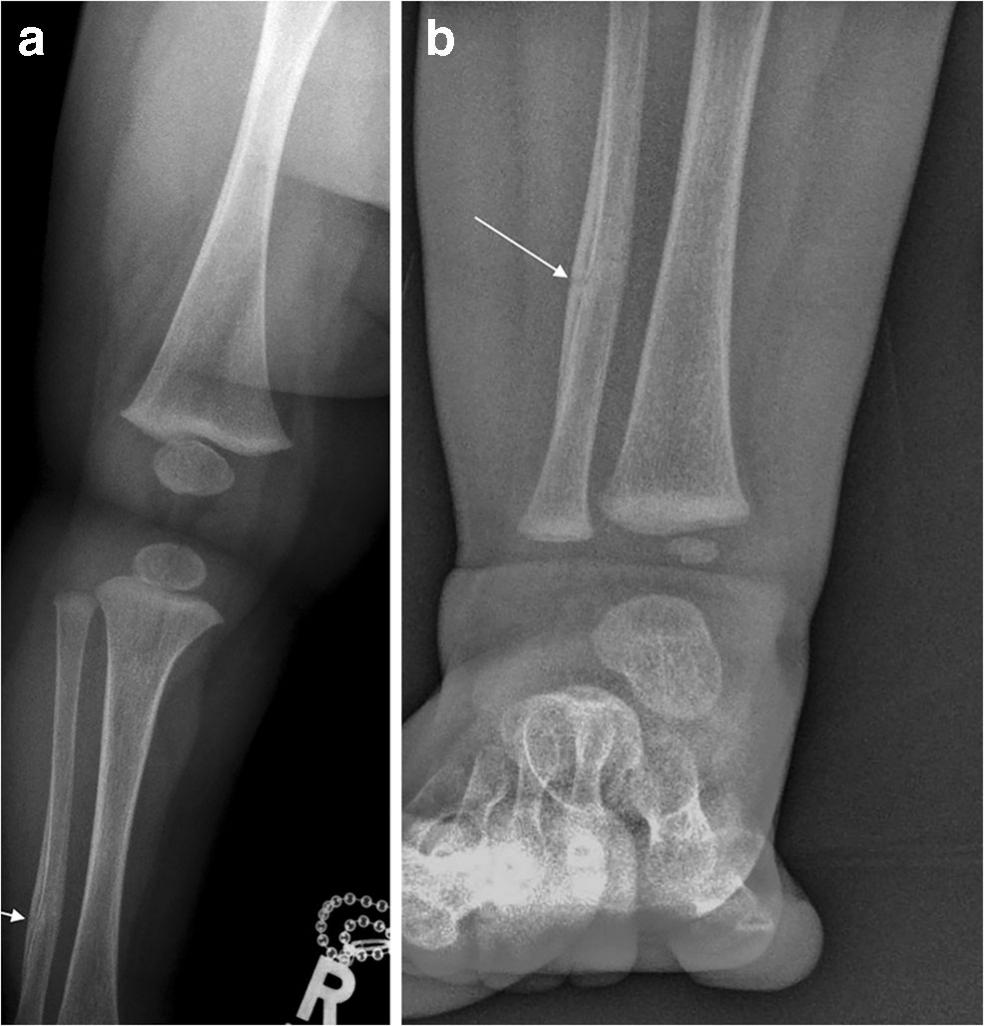
Right leg (**a**) and ankle (**b**) anteroposterior projections taken as part of the initial skeletal survey, 3 days after the images in Fig. 1 and a day after that in Fig. 2. There is a healing undisplaced fracture of the right fibular shaft at the junction of middle and distal thirds (*arrows*), i.e. at an almost identical location as the fracture of the left fibula seen in Figs. 1 and 2

**Fig. 4:**
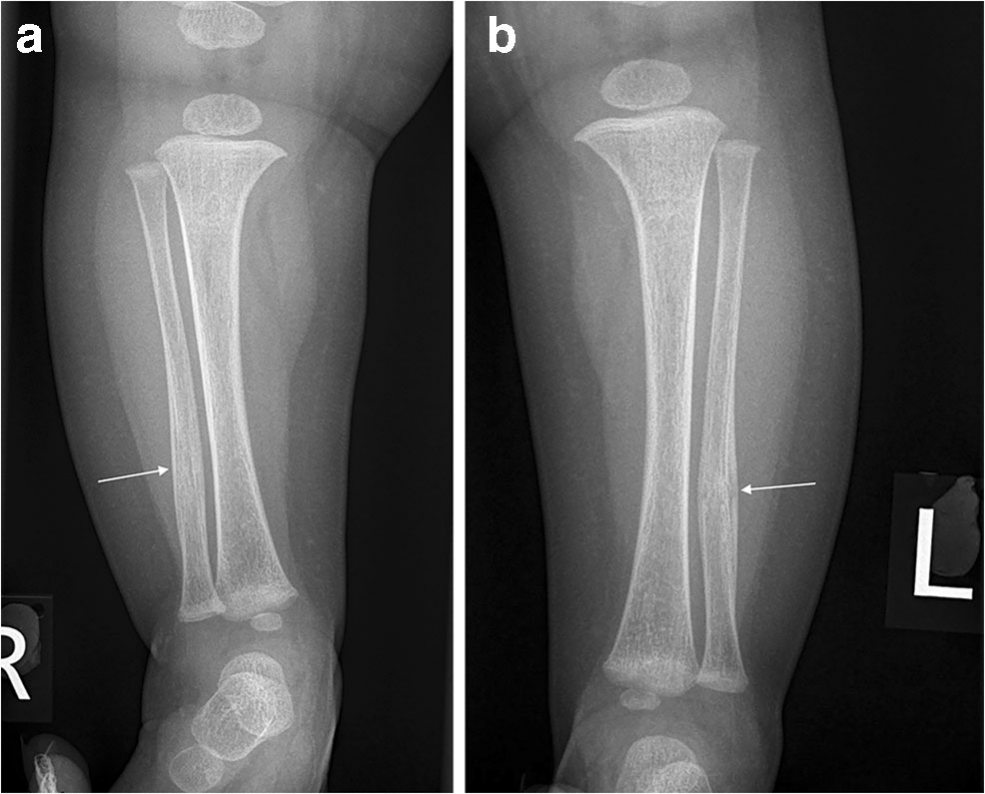
Right (**a**) and left (**b**) anteroposterior projections of the tibiae and fibulae taken 5 weeks after those in Fig. 3. There is progressive healing of the left tibial fracture (*arrow*), whilst the fracture on the right has completely healed (*arrow*)

Anteroposterior and lateral radiographs of the left leg demonstrated a subtle undisplaced fracture of the left fibula but were initially reported as normal (Fig. 1). An orthopaedic follow-up radiograph (Fig. 2) performed 2 days later because of persistent symptoms demonstrated increased periosteal reaction and the suspicion of inflicted injury was raised. An initial skeletal survey (excluding the left leg) revealed a further healing undisplaced fracture of the right fibular diaphysis (Fig. 3) at an almost identical position to the left fibular fracture. The finding of healing bilateral fibular fractures was reported as unusual, further raising the suspicion of inflicted injury. A follow-up skeletal survey was not performed. However, by the time of repeat radiographs 5 weeks later, the right fracture had healed, while the left showed evidence of further interval healing (Fig. 4).

Radiographic bone modelling and density were normal with no features to indicate an underlying disorder that might predispose the patient to fracture. Bone profile and vitamin D (117.3 nmol/L) were normal and did not suggest bone fragility. The head computed tomography and ophthalmology examinations were normal.

**Fig. 5:**
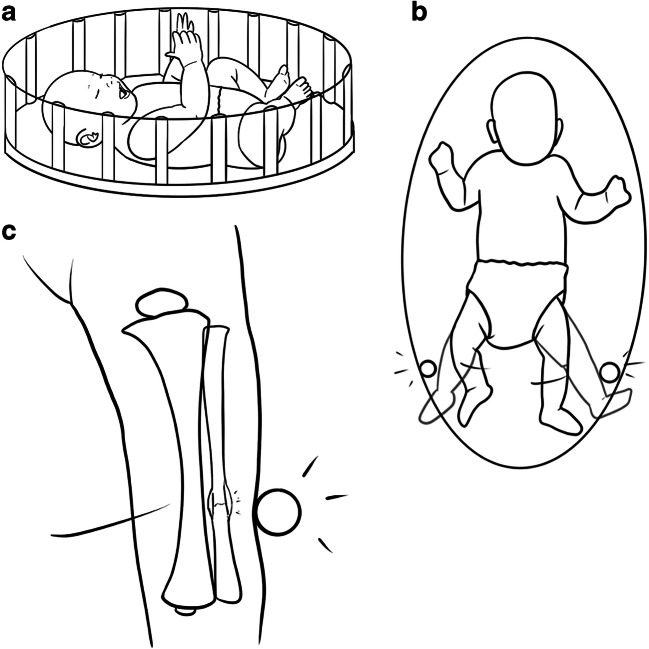
An illustration of the mechanism of injury demonstrated in the videos provided by the parents to explain the healing bilateral fibular fractures in Figs. 1, 2, 3 and 4. **a** Side view of the 6-month-old boy in his playpen with surrounding metal bars. **b** Bird’s eye view of (**a**), which demonstrates that the lateral aspects of the infant’s legs repeatedly hit the metal frame of the playpen at approximately the same level at which the radiologic abnormalities were identified. Only one set of metal bars is illustrated. **c** An anteroposterior view of the left leg from (**b**) demonstrates the left fibula and overlying muscle hitting the metal bar, which over prolonged periods of time result in stress fracture. Only the left lower limb is illustrated

Legal proceedings commenced, during the course of which it occurred to the child’s parents that the fractures might have been sustained as a result of repeatedly banging his legs against the metal frame of his playpen. They were consistent in providing this explanation and produced several videos to illustrate the mechanism (Fig. 5). A review of the videos by the radiology expert instructed in the matter confirmed that the lateral aspects of the infant’s legs repeatedly hit the metal frame of the playpen over prolonged periods at approximately the same level at which the radiologic abnormalities were identified. Given that the fractures of both fibulae occurred at near identical positions with no other acute or healing radiologic or clinical injury identified, it was believed that on the balance of probabilities, this purported mechanism of repeated low-energy impact force was consistent with the injuries sustained.

## Discussion

Fibular fractures are rarely seen in physical abuse – when they do occur, they result from direct impact to the fibular shaft and typically alongside a tibial fracture [3] — or if undisplaced, they may result from indirect forces as the leg is bent or twisted. Stress fractures result from repetitive low-grade forces, each insufficient to cause a fracture but cumulatively weaken both the bone and the overlying muscle, eventually leading to fracture. Fibular stress fractures result from repetitive injuries, usually in ambulant athletic younger children and adults: It is thought that in toddlers, they result from the novel stresses associated with new/developing ambulation [4].

Abusive fractures are more common in children younger than 2 years old. Half of all fractures in infants younger than 12 months old are attributable to physical abuse [5] with the highest incidence at 4 months of age [6]. Multiple long-bone fractures, especially bilateral fractures, are of moderate specificity for abuse. The finding of bilateral isolated healing fibular fractures in a pre-ambulant 6-month-old infant is suspicious for inflicted injury by virtue of fracture location and multiplicity and patient age and pre-ambulatory status. Bilateral healing fibular fractures in a non-ambulant child, i.e. a child with a permanent physical disability or totally dependent child who will never be able to walk, also raises the suspicion of inflicted injury. Indeed, any fracture with an inappropriate history is suspicious. Inflicted injury and other conditions that predispose to fracture should be excluded, particularly given that neither of the identified fractures (Figs. 1, 2 and 3) will have been sustained from normal day-to-day handling of an infant this age. There was no radiologic or serological evidence of metabolic bone disease, osteogenesis imperfecta or other cause of propensity to fracture.

This fracture pattern has been reported in a 10-month-old girl following repetitive banging of the child’s walker (supported ambulation) against a kitchen cabinet with her lower legs at the level of the fractures [7]. They have also been described in a 26-month-old boy, for whom there was no history of trauma or other apparent explanation [4]. We acknowledge that there is no proof of our proposed hypothesis. In an infant of this age, a personalised biomechanical and/or finite element testing of this mechanism would be required, similar to a recently published study investigating rolling as a mechanism for humeral fractures in non-ambulant infants [8].

Where radiologic evidence of bony injury in infants and young children has been identified, it must be considered within the clinical context in which it is presented, and a determination made as to whether any proffered history or mechanism of injury could account for the injury. The objective evidence of multiple episodes of sustained repetitive banging of our patient’s legs against the metal frame of his playpen was accepted as the causative mechanism of injury. It is vital that physical abuse is excluded as the cause of injury, as a misdiagnosis risks leaving a child with abusive parents or caregivers. Other less common non-inflicted causes of otherwise unexplained fractures must also be considered to avoid an incorrect diagnosis of physical abuse and erroneously separating a child from their parents or caregivers. Both scenarios are potentially damaging to children and their caregivers. Radiologic imaging constitutes only part of the investigation of suspected physical abuse and clinicians must ensure that an overview of the clinical picture is presented to formulate a reasoned diagnosis based on all the available evidence.
